# Smartphone and Internet Access and Utilization by People With Schizophrenia in South Australia: Quantitative Survey Study

**DOI:** 10.2196/11551

**Published:** 2020-01-28

**Authors:** Kwok Tung Gordon Wong, Dennis Liu, Ryan Balzan, Daniel King, Cherrie Galletly

**Affiliations:** 1 The University of Queensland Brisbane Australia; 2 The University of Adelaide Discipline of Psychiatry, Adelaide Medical School Adelaide Australia; 3 Northern Adelaide Local Health Network Adelaide Australia; 4 The University of Adelaide School of Psychology Adelaide Australia; 5 Flinders University College of Education, Psychology and Social Work Adelaide Australia; 6 Ramsay Health Care (SA) Mental Health Services Adelaide Australia

**Keywords:** schizophrenia, schizoaffective disorder, internet, technology, computer, smartphone

## Abstract

**Background:**

Web-based information and interventions for mental illness are increasingly being provided. There is an expectation that citizens have access to the internet and are competent in using technology. People with schizophrenia are often excluded from social engagement, have cognitive impairment, and have very limited income, all of which may reduce their use of technology.

**Objective:**

This study aimed to investigate technology access, use of digital technology, and confidence in using technology among people with schizophrenia living in the community.

**Methods:**

Face-to-face structured interviews with 50 people with schizophrenia (aged 18-65 years) living in the northern suburbs of Adelaide, South Australia, were conducted using an instrument designed to assess technology access and utilization.

**Results:**

Most participants (42/50, 84%) owned a mobile phone, but only 58% (29/50) owned a smartphone. Two-thirds of participants (33/50, 66%) had access to the internet at home, using a smartphone or computer. Moreover, 40% (20/50) of participants used the internet at least daily, but 30% (15/50) of participants had never accessed the internet from any device. Approximately half of the participants (24/50, 48%) had never used Facebook. Participants rarely used community facilities (eg, libraries and cafes) to access the internet. There were no significant differences (*P* values ranged from .14 to .70) between younger participants (aged 18-34 years) and older participants (aged 35-64 years) in internet or smartphone access or confidence in using technology.

**Conclusions:**

Although the sample size of this study is small, it shows limited technology access, use of digital technology, and confidence in using technology among the participants. This could be a barrier to the online delivery of information and interventions for people with schizophrenia. To better understand the impacts of such technological disadvantage and potential disparities in access and use of online resources, prospective studies should recruit a larger sample size and include control subjects matched for socioeconomic disadvantage.

## Introduction

### Background

Electronic mental (e-mental) health interventions are increasingly being implemented to assist in the management of psychiatric disorders and improve access to early intervention [1,2]. Those interventions apply information and communication technology, eg, computers and smartphones, to help promote mental well-being [1]. Although many of these interventions are aimed at high-prevalence mental health issues, eg, anxiety and depression [3], some specifically target people with psychotic disorders [4]. Emerging research suggests that engagement with cost-effective online interventions may be feasible for adults with a schizophrenia spectrum disorder [5]. For example, Alvarez-Jimenez et al [4] used an online platform to deliver clinical interventions to young people with early schizophrenia. Montes et al [6] found that the use of SMS text messages can improve antipsychotic medication adherence in people with schizophrenia, and studies by Ainsworth et al [7] and Ben-Zeev et al [8] demonstrated that smartphones could be beneficial in delivering interventions to patients with schizophrenia.

A systematic review by Alvarez-Jimenez et al [5] found that online, social media, and mobile phone–based interventions show promise in reducing the severity of positive symptoms and depression, reducing hospital admissions, improving social contacts, and enhancing medication adherence. There has been limited research on technology access (such as computers and smartphones), use of digital technology, and the level of confidence in using technology among individuals with schizophrenia. Access to technology is fundamental for the implementation of e-mental health.

In addition to potentially limiting access to e-mental health interventions, limited access to and use of technology could be associated with low levels of workforce engagement [9,10], reduced access to essential services (eg, government departments and financial institutions) [10] and transport systems [11], and limited access to social media [12]. Those factors could further impair social exclusion.

Miller et al [12] surveyed 80 inpatients and outpatients with schizophrenia at a university psychiatry service in the United States. They found that 56% of patients used text messaging, 48% had an email account, and 27% used social networking sites regularly. Gay et al [13] reported that of 457 people with schizophrenia, 24% used Web-based technology to help identify coping strategies, 42% listened to music to help block or manage voices, and 28% used technology to set alarms or reminders for medication management. However, Gay et al [13] used an online survey to collect this information; therefore, their sample was biased to those comfortable with online activities and did not include people who lacked internet access or competence using technology. A propensity weighting method was used to adjust for socioeconomic, attitudinal, and behavioral differences between people who respond to Web-based surveys and those who do not, but this appeared to be based on the general population rather than taking into account specific differences between those with schizophrenia and the rest of the community. Torous et al [14] reported substantial differences according to socioeconomic status. Only 33% of outpatients attending a state mental health clinic in the United States owned a smartphone and were willing to use it to monitor psychiatric symptoms, compared with 72% of those attending a private clinic [14]. Glick et al [15] surveyed 100 people with severe mental illness attending a public sector community mental health center in Atlanta, United States. They found that 85% of their participants had a cell phone and 37% had a smartphone, compared with 53% in a general population survey, and 44% of the socioeconomically disadvantaged people in the general community had smartphones. Both Torous et al [14] and Glick et al [15] surveyed outpatient populations that included a range of psychiatric diagnoses. In the study by Glick et al [15], 29.2% of people had schizophrenia or schizoaffective disorder, and Torous et al [14] did not include diagnostic information. Similarly, Ennis et al [9] drew participants from an early psychosis unit and from community mental health services in London. They did not give detailed diagnostic information. They found that technology use and access were similar to the general population. Collectively, those studies about the access to technology and the use of technology in patients with a schizophrenia spectrum disorder appeared inconclusive, which suggests a need for further investigation in those areas. Nonetheless, those findings correspond to the findings by Firth et al [16] who showed that smartphone app–based interventions for the whole population of patients with schizophrenia are not feasible when a significant portion of these people have no access to a smartphone. Together, those studies have justified the need to further investigate and understand their technology access and use, rather than implementing e-mental health interventions in the said population.

There are some specific aspects of schizophrenia that would be expected to be relevant in this study’s context. People with schizophrenia generally have some cognitive impairment [17], which would impact the ability to learn new skills using unfamiliar devices. Negative symptoms (eg, avolition and poor initiative), lack of social networks [18], and paranoid ideation [12] may also reduce engagement with technology. Furthermore, most people with psychotic disorders have very limited income [19]; hence, the purchase of smartphones and computers may not be possible. Libraries generally have computers with internet access that can be used by the general public, and internet access is available in many cafes, yet the usage of these low-cost options by people with schizophrenia is not known.

This study took place in the northern suburbs of Adelaide, a region with a low socioeconomic status. The official rate of unemployment is 7.7%, which is higher than that in other metropolitan areas in South Australia. Almost two-thirds (62%) of the population had not completed secondary school education [19], and in 2016, more than three-quarters (77%) of the northern population had received government benefits for more than 1 year [20]. The health status of people residing in this region is generally poorer than that of people residing in other regions in South Australia, with one-quarter of residents (25%) reported to have a serious or chronic health condition [21].

### Objectives

The first objective of this study was to investigate technology access, use of digital technology, and confidence in using technology in people with schizophrenia living in a socioeconomically deprived urban region in Adelaide, Australia. The second objective was to compare internet usage, frequency of internet use, and confidence in using technology in younger and older adults with schizophrenia.

## Methods

### Recruitment

For this quantitative survey study, a member of the research team, psychiatrist (DL), identified stable outpatients with schizophrenia attending the Northern Adelaide Local Health Network (NALHN) community mental health center who were interested in participating in research. Afterward, another research team member (GW) recruited them for the study.

A total of 51 adults agreed to participate, but 1 of them was then found to have a diagnosis other than schizophrenia or schizoaffective disorder, resulting in the final sample of 50 adults. On participation, each participant was given an Aus $20 shopping gift card to reimburse their time. Owing to the budget allocated for this research project, only 50 adults with schizophrenia were recruited.

### Participants and Exclusion Criteria

To ensure that participants had the capacity to consent for participation, individuals with a history of moderate/severe head injury, neurological disorders, or mental retardation (ie, IQ<70 as determined by the National Adult Reading Test [NART] [22]) were excluded.

### Survey

Two of the research team members (GW and CG, a psychiatrist) were responsible for the conceptualization and selection of this study’s survey constructs, based on professional experiences in working with outpatients with schizophrenia. In addition to the NART, this study’s interview consisted of Peters et al Delusions Inventory 21 [23], which measures delusion proneness. There were also items of demographic information and mental health history developed from a recent Australian study, People Living with Psychotic Illness 2010 [19]. Moreover, 80 individual technology-related questions that were used in this study were modified from surveys developed by Ennis et al [9], Muñoz-Neira et al [24], and Miller et al [12]. These questions assessed the following domains: (1) access, prevalence, and frequency of use of computers and mobile phones in general; (2) use of online resources (eg, banking, navigation, online shopping, email, social media, and well-being); and (3) attitudes to technology and self-rated competence.

For further details about this study’s survey, please see Multimedia Appendix 1.

### Procedure

**Figure 1:**
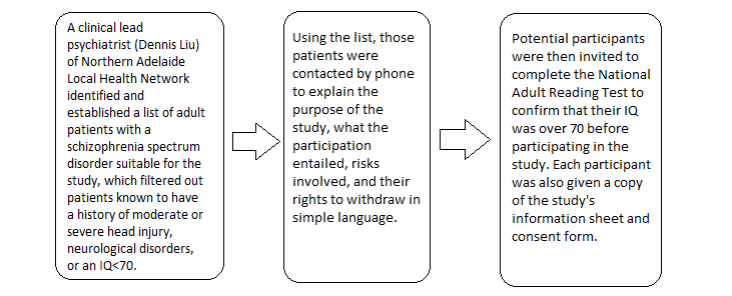
Recruitment flow-chart for this study.

The Human Research Ethics Committee of The Queen Elizabeth Hospital, Adelaide, South Australia, approved this research project. Before participation, GW explained to each participant the purpose of the study, what the participation entailed, the potential risks involved, and that they were free to withdraw without prejudice. Each participant was also given a copy of the study’s participant information sheet and consent form. All participants gave written informed consent. The recruitment flowchart is shown in Figure 1. Individual interviews were conducted in the NALHN mental health sites, including a hospital-based clinical trials unit and a psychosocial rehabilitation and day program club, by a community rehabilitation worker (GW) under supervision. Instead of asking the study’s participants to fill out the study questionnaire themselves, the primary researcher (GW) interviewed the participants as a means to gauge their understanding of the questionnaire questions. The interviews lasted for 45 to 60 min each.

### Data Analysis

Chi-square tests were used to investigate whether younger participants (aged 18-34 years) had better technology access and more confidence in using technology than older participants (aged 35-64 years).

## Results

### Demographics

Participants were predominantly male with schizophrenia and were supported by government pensions. Moreover, 30% (15/50) of the participants were aged between 18 and 34 years, 30% (15/50) were aged between 35 and 44 years, and another 30% (15/50) were aged between 45 and 54 years. The remaining participants (5/50, 10%) were aged between 55 and 65 years (Table 1). In addition, 40% (20/50) of the participants had completed secondary school education; 52% (26/50) of the participants used Facebook, and their mean number of friends on Facebook was 52.86 (SD 114.58). Most participants (43/50, 86%) had been in contact with mental health services for 7 years or more, and 94% (47/50) of the participants took atypical antipsychotic medication.

### Ownership of Devices and Usage

Overall, 42 participants (42/50, 84%) owned a mobile phone, but only 29 (29/50, 58%) owned a smartphone. In addition, 26 participants (26/50, 52%) owned a personal computer, and 14 participants (14/50, 28%) owned a tablet. Half of the participants owned more than 1 device. Table 2 shows the rates of use of technology, with 76% (38/50) using their devices at least weekly.

Regarding smartphone use, only 44% (22/50) of the participants had ever downloaded an app, and 38% (19/50) of the participants used GPS or navigated using maps; in contrast, the majority of the participants had used their device’s calendar (35/50, 70%), calculator (33/50, 66%), alarm (30/50, 60%), and camera (31/50, 62%). Only 32% (16/50) of the participants used their computers for more complex tasks such as word processing.

**Table 1:** Sociodemographic and clinical description of the sample (N=50).

Characteristics			Value
**Gender, n (%)**			
	Male	31 (62)	
	Female	19 (38)	
**Age (years), n (%)**			
	18-34	15 (30)	
	35-44	15 (30)	
	45-54	15 (30)	
	55-65	5 (10)	
**Highest education completed, n (%)**			
	Did not complete year 9	3 (6)	
	Did not complete secondary school (year 12), no postsecondary school qualifications	25 (50)	
	Secondary school certificate	7 (14)	
	Postschool qualification (trade certificate or higher)	13 (26)	
	Unsure	1 (2)	
	Did not answer	1 (2)	
**Income (some had more than 1 source of income), n (%)**			
	Government pension	50 (100)	
	Full-time work	1 (2)	
	Part-time work	5 (10)	
	Casual work	2 (4)	
	Financial support from family	14 (28)	
	Financial support from other people	3 (6)	
**Diagnosis, n (%)**			
	Schizophrenia	43 (86)	
	Schizoaffective disorder	7 (14)	
**Duration of involvement with mental health services, n (%)**			
	1 to <3 years	1 (2)	
	3 to <5 years	1 (2)	
	5 to <7 years	5 (10)	
	≥7 years	43 (86)	
	Admitted to a mental health unit in the last 12 months	24 (48)	
**Medication, n (%)**			
	Typical antipsychotic medication	4 (8)	
	Atypical antipsychotic medication	47 (94)	
	Antidepressant medication	22 (44)	
	Antianxiety medication	2 (4)	
	Anticholinergic medication	2 (4)	
**Peters et al Delusions Inventory 21, mean (SD)**			
	Total score	70.26 (13.67)	
	Yes/no score	7.32 (4.55)	
	Distress score	20.18 (15.89)	
	Preoccupation score	20.40 (15.81)	
	Conviction score	22.32 (16.24)	
**Intelligence score, mean (SD)**			
	Premorbid IQ (Wechsler Adult Intelligence Scale-Revised) score	103.06 (13.67)	

**Table 2:** Access to technology (N=50).

Level of frequency	The use of any technology devices, n (%)	The use of internet, n (%)	Computer access at library or community center, n (%)	Wi-Fi access at library or cafe with smartphone, n (%)	Wi-Fi access at library or cafe with laptop, n (%)	The use of Facebook, n (%)	The use of email, n (%)	The use of internet banking, n (%)	Making online payments, n (%)	Engaging in self-development study activities online, n (%)	Looking and applying for jobs or studies online, n (%)	Frequency of using the internet to look up health-related information, n (%)
Never	9 (18)	15 (30)	34 (68)	43 (86)	48 (96)	24 (48)	19 (38)	28 (56)	33 (66)	35 (70)	33 (66)	27 (54)
Less than monthly	1 (2)	3 (6)	8 (16)	4 (8)	2 (4)	4 (8)	4 (8)	3 (6)	6 (12)	6 (12)	6 (12)	6 (12)
Monthly	0 (0)	3 (6)	5 (10)	2 (4)	0 (0)	4 (8)	6 (12)	3 (6)	4 (8)	4 (8)	3 (6)	11 (22)
Fortnightly	1 (2)	0 (0)	0 (0)	0 (0)	0 (0)	0 (0)	0 (0)	2 (4)	0 (0)	0 (0)	0 (0)	0 (0)
Weekly	1 (2)	3 (6)	1 (2)	0 (0)	0 (0)	0 (0)	3 (6)	0 (0)	3 (6)	0 (0)	0 (0)	0 (0)
More than weekly	5 (10)	6 (12)	2 (4)	1 (2)	0 (0)	2 (4)	4 (8)	9 (18)	3 (6)	3 (6)	6 (12)	2 (4)
Daily	20 (40)	12 (24)	0 (0)	0 (0)	0 (0)	12 (24)	10 (20)	5 (10)	1 (2)	2 (4)	1 (2)	4 (8)
More than daily	13 (26)	8 (16)	0 (0)	0 (0)	0 (0)	4 (8)	4 (8)	0 (0)	0 (0)	0 (0)	1 (2)	0 (0)

#### Internet Access

Two-thirds (33/50, 66%) of the participants had access to the internet at home, using a smartphone, computer, or both. In addition, 40% (20/50) of the participants used the internet at least daily (Table 2). However, 30% (15/50) of the participants had never accessed the internet from any device. Community facilities, such as cafes, libraries, and community centers, which provide internet access, were rarely used for this purpose by the participants in this study.

Of the participants using the internet, 44% (22/50) used it for internet banking and 30% (15/50) to 34% (17/50) used it for online shopping, for making online payments, for applying for jobs or courses online, or for engaging with self-development activities. Only half of the participants with internet access used the internet for searching for local businesses (24/50, 48%) or to plan trips (25/50, 50%). Moreover, 54% (27/50) of the participants never used the internet to look up health-related information online, and 38% (19/50) of the participants reported that they could not find an Australian website about depression.

#### Digital Communication

Most participants (44/50, 88%) used mobile phones at least weekly to make and receive calls, with a lower proportion (32/50, 64%) using text messaging, and 38% (19/50) never used email. Social media was not routinely used, and 48% (24/50) and 90% (45/50) of the participants had never used Facebook and Twitter, respectively, on any device.

#### Attitudes to Technology

About 1 in 3 participants were not confident with using smartphone devices (Table 3). Fewer than half of the participants reported that they would have trouble with their day-to-day life if they did not have access to the internet (22/50, 44%), computer (22/50, 44%), or their smartphone devices (21/50, 42%).

A higher percentage of people aged 18 to 34 years used the internet at home (11/15, 73%), used the internet daily (7/15, 47%), and had greater confidence in using a computer or smartphone (60%) compared with people aged 35 to 64 years (22/35, 63%; 13/35, 37%; and 29% respectively). However, these differences between the 2 age groups were not statistically significant (Table 3).

**Table 3:** Internet usage, internet frequency, and confidence in using technology by age (N=50).

Internet usage, internet frequency, and confidence in using technology by age			18-34 years (n=15), n (%)		35-64 years (n=35), n (%)		Chi-square (*df*)		*P* value
**Use internet on computer or mobile phone at home**						0.2 (1)		.70	
	No	4 (27)		13 (37)					
	Yes	11 (73)		22 (63)					
**Frequency of internet use**						2.4 (3)		.50	
	Never	3 (20)		12 (34)					
	Monthly or less	1 (7)		5 (14)					
	Weekly	4 (27)		5 (14)					
	Fortnightly	0 (0)		0 (0)					
	Daily	7 (47)		13 (37)					
**Confidence in using computer**						1.2 (2)		.56	
	Not at all confident	6 (40)		13 (37)					
	Somewhat confident	3 (20)		12 (34)					
	Confident or very confident	6 (40)		10 (29)					
**Confidence in using smartphone**						3.9 (3)		.14	
	Not at all confident	5 (33)		11 (31)					
	Somewhat confident	1 (7)		11 (31)					
	Confident or very confident	9 (60)		13 (37)					

## Discussion

### Principal Findings

Overall, technology access and engagement were lower in this study than in the rest of the Australian community. The prevalence of owning a computer (26/50, 52%) or smartphone (29/50, 58%) in our participants was lower than that in the Australian population (62% and 76%, respectively) [25]. Similarly, in 2016, it was estimated that 87% of Australians used the internet daily [26], compared with 40% (20/50) of our participants with schizophrenia, and 50% of Australians used social networking sites daily, compared with 32% (16/50) in our sample. Approximately 30% (15/50) of our sample had never accessed the internet.

Our results showed that most participants were only using certain device functions (eg, calling and SMS text messaging) and rarely used other functions of digital technology (eg, internet banking, social networking, self-development, health maintenance, and navigation). There was little use of e-mental health therapeutic interventions or psychoeducational sites.

Some studies provide smartphones to enable participants in research projects using e-mental health interventions. In contrast, participants in other research projects are required to use their own smartphone. Treisman et al [25] attributed the lack of engagement with technology by some people with schizophrenia to the cost of devices and lack of skills. Glick et al [15] argue that smartphones and internet plans are becoming affordable, but people with psychoses often describe living in poverty. In an Australian survey in 2010, 12% of the study sample reported having run out of food in the previous 12 months, and not having money to buy more [12]. In addition, it is possible that the costs associated with a technology device [27], a lack of knowledge about what device to buy, and how to set up internet access may be barriers to use. Furthermore, cognitive impairment is a core symptom of schizophrenia [28,29], so learning to use unfamiliar devices and to acquire technology skills could be challenging.

Increasingly, social engagement occurs via social media, and information from a wide variety of sources is provided online rather than using more traditional methods of communication. Many people with psychosis, including schizophrenia, experience social isolation [30,31], potentially with a lack of meaningful social participation. It appears that one-third of the participants would be unable to engage in social media. However, less than half of the participants showed that they would never struggle with their daily life if they had no access to the internet, computer, or their smartphone. Although the rationale was not investigated, such phenomena could be related to an adaptation to live without those technologies or the individual’s regular psychosocial rehabilitation support, which may include side-by-side assistance for activities of daily living offered by mental health support workers and clinicians, reducing the need to use technologies independently.

This study provides data on the level of access to digital technology and the self-reported confidence among adults with schizophrenia living in a socioeconomically deprived urban region in Australia. These findings may have implications for the design and scope of delivery of e-mental health interventions for this particular population, especially when there are e-mental health interventions that target psychoeducation (via the internet) and medication adherence (via SMS text messages) [32]. Yet, using e-mental health interventions requires a certain level of technology access, use, and confidence. When patients with schizophrenia do not use digital technology or lack the confidence to use it, they may not be able to fully use those e-mental health interventions.

### Future Directions

Prospective studies may need to address barriers that prevent greater engagement with technology in this population, including paranoia [12], mistrust of devices, and access to the internet. Further research could also examine the barriers to accessing the internet in public areas (eg, public libraries), given that these resources were rarely used. Needless to mention that, in the NALHN’s service catchment area, at least three libraries with free internet access and training are located within walking distances to the corresponding major shopping centers and supermarkets. This is particularly concerning, as the cost of purchasing devices and maintaining internet access may simply be too expensive for many people with schizophrenia, and these free services may be a useful means of access. Specific familiarization programs are needed to help with awareness of these facilities and with feeling welcome when attending. Ultimately, improved access and encouraging patients with schizophrenia to learn how to use technology may promote a greater range of technology uses, potentially including therapeutic interventions.

### Limitations

The small sample size of this study limits generalizability. In addition, instead of assessing real-time usage and skills as a technology literacy assessment, this study relied on self-reported frequency of technology usage. This study did not assess technology literacy. In other words, this study did not capture the extent to which participants could carry out a task, eg, asking them to navigate a website, perform a search, and note any difficulties they encounter. Nonetheless, this research has tapped into the participants’ confidence level regarding technology use. Future research could also include an economically and demographically matched healthy control group to more thoroughly examine factors unique to individuals with schizophrenia.

### Conclusions

In conclusion, digital technology use and internet access were limited in this population. This may be addressed via the delivery of training to increase individuals’ confidence in the use of technology. This training may be required before the online delivery of e-mental health interventions can be widely used in people with schizophrenia. Further research should examine the extent to which paranoia, cognitive deficits, and poverty limit people with schizophrenia from engaging with e-mental health interventions, smartphones, and computers to improve their mental health.

## Appendix

Multimedia Appendix 1 Demographic information.

## Abbreviations

e-mental: electronic mental
NALHN: Northern Adelaide Local Health Network
NART: National Adult Reading Test
