# Supplementary material for: Smartphone and Internet Access and Utilization by People With Schizophrenia in South Australia: Quantitative Survey Study
Source: JMIR Ment Health. 2020 Jan 28;7(1):e11551. doi: 10.2196/11551 (PMC7013647; doi:10.2196/11551)
Supplement: Multimedia Appendix 1 [file mental_v7i1e11551_app1.docx]

**Appendix**

**Demographic information**

Your age: О 18 – 24 О 25 – 34 О 35 – 44 О 45 – 54

О 55 – 64

Your sex: О Male or О Female

Your highest education qualification level:

О Did not complete Year 9.
О Did not complete Year 12, no post school qualification.
О Year 12 Certificate.
О Post school qualification (trade certificate or higher).
О I’d rather not to answer.
О I am not sure.

What is your primary diagnosis?

__________________________________

How long have you been involved with mental health services?

О Less than 1 year
О 1 to less than 3 years
О 3 to less than 5 years
О 5 to less than 7 years
О 7 years or more

In the last 12 months, how many admissions have you had to a mental health unit?

О 0 to 2 times
О 3 to 5 times
О 6 to 8 times
О 9 times or more

How long was your inpatient psychiatric treatment in the last 12 months?

О Less than 2 weeks.
О 2 to less than 4 weeks.
О 4 weeks to less than 13 weeks.
О 13 weeks to less than 26 weeks.
О 26 weeks to 52 weeks.

What is your current source of income? (Tick one or more)

О Government pensions.
О Full-time employment.
О Part-time employment.
О Casual employment.
О Financial support from your family.
О Financial support from other people.

What prescribed medication did you use in the last four weeks to address your mental health problems?

____________________________________

____________________________________

____________________________________

____________________________________

**Part B – Technology literacy**

**Section I – Technology-related background information**

What types of information communication technology devices do you have? (Tick one or more)

О Microsoft Windows Desktop and/or laptop.
О Apple Macbook and/or iMac.
О Apple iPad.
О Apple iPhone.
О Android smartphone.
О Android tablet.
О Blackberry smartphone.
О Others. Please specify:_________________________.

How often do you use the information communication technology devices mentioned above?

0 1 2 3 4 5

Never Less than Monthly More than Daily More than monthly weekly daily

Apart from computer, what types of standalone gaming console do you have? (Tick one or more)

О Microsoft Xbox 360.
О Microsoft Xbox One.
О Sony Playstation 3.
О Sony Playstation 4.
О Nintendo Wii.
О Others. Please specify:_________________________.

How confident are you when it comes to using a smartphone in general? (Please circle the corresponding number.)

 0 1 2 3

Not at all Somewhat Confident Very
 confident confident confident

How confident are you when it comes to using a computer in general? (Please circle the corresponding number.)

 0 1 2 3

Not at all Somewhat Confident Very
 confident confident confident

Section II – Basic access to internet

How often do you use the internet?

 0 1 2 3 4 5

Never Less than Monthly More than Daily More than

monthly weekly daily

Are you able to tell if your technology devices are connected to the internet or not?

О Yes О No

Do you have internet access at home?

О Yes О No

How often do you go to a library or a community centre to use their computers?

0 1 2 3 4 5

Never Less than Monthly More than Daily More than
 monthly weekly daily

How often do you go out to use Wi-Fi, e.g., in a library or at a café, using your own smartphone?

0 1 2 3 4 5

Never Less than Monthly More than Daily More than

monthly weekly daily

How often do you go out to use Wi-Fi, e.g., in a library or at a café, using your own laptop?

0 1 2 3 4 5

Never Less than Monthly More than Daily More than

monthly weekly daily

Do you share the internet connection of your phone or tablet with other devices?

О Yes О No

Do you use a standalone internet dongle?

О Yes О No

**Section III - Finance**

How often do you use ATMs?

0 1 2 3 4 5

Never Less than Monthly More than Daily More than

monthly weekly daily

How often do you use internet banking?

0 1 2 3 4 5

Never Less than Monthly More than Daily More than

monthly weekly daily

How often do you shop online?

0 1 2 3 4 5

Never Less than Monthly More than Daily More than

monthly weekly daily

How often do you make payments online?

0 1 2 3 4 5

Never Less than Monthly More than Daily More than

monthly weekly daily

**Section IV – Lifestyle and relaxation**

Do you play or have you ever played video games? [If so, please administer Gaming Cognition Scale at the end of this interview]

О Yes О No

How often do you play games on your tablet, computer, Microsoft Xbox, Sony PlayStation, Nintendo Wii, without being connected to the internet?

0 1 2 3 4 5

Never Less than Monthly More than Daily More than

monthly weekly daily

How often do you play online games (connected to the internet)?

0 1 2 3 4 5

Never Less than Monthly More than Daily More than

monthly weekly daily

How often do you play games on your phone/smartphone?

0 1 2 3 4 5

Never Less than Monthly More than Daily More than

monthly weekly daily

How often do you listen to music online?

0 1 2 3 4 5

Never Less than Monthly More than Daily More than

monthly weekly daily

How often do you listen to radio channels online?

0 1 2 3 4 5

Never Less than Monthly More than Daily More than

monthly weekly daily

How often do you watch videos online, e.g., YouTube?

0 1 2 3 4 5

Never Less than Monthly More than Daily More than

monthly weekly daily


Section V – Online social networking and communication

How often do you use facebook or other social media websites?
0 1 2 3 4 5

Never Less than Monthly More than Daily More than

monthly weekly daily

If you use facebook, how many facebook friends do you have?

______________________________

How often do you use twitter?

0 1 2 3 4 5

Never Less than Monthly More than Daily More than

monthly weekly daily

If you use twitter, how many followers do you have?

______________________________

If you use twitter, how many people do you follow on twitter?

______________________________

How often do you use email?

0 1 2 3 4 5

Never Less than Monthly More than Daily More than

monthly weekly daily

How often do you check your email?

0 1 2 3 4 5

Never Less than Monthly More than Daily More than

monthly weekly daily

How often do you open and read email attachments?

0 1 2 3 4 5

Never Less than Monthly More than Daily More than

monthly weekly daily

How often do you reply to emails?

0 1 2 3 4 5

Never Less than Monthly More than Daily More than

monthly weekly daily

How often do you receive emails from your doctors?

0 1 2 3 4 5

Never Less than Monthly More than Daily More than

monthly weekly daily

How often do you send emails to your doctors?

0 1 2 3 4 5

Never Less than Monthly More than Daily More than

monthly weekly daily

How often do you make video calls, e.g., Skype or FaceTime?

0 1 2 3 4 5

Never Less than Monthly More than Daily More than

monthly weekly daily

**Section VI – Basic functions**

How often do you use your mobile phone’s calendar?

 0 1 2 3 4 5

Never Less than Monthly More than Daily More than

monthly weekly daily

How often do you use your mobile phone’s calculator?

0 1 2 3 4 5

Never Less than Monthly More than Daily More than monthly weekly daily

How often do you use your mobile phone’s alarm?

0 1 2 3 4 5

Never Less than Monthly More than Daily More than monthly weekly daily

How often do you send/receive text messages with mobile phone?

0 1 2 3 4 5

Never Less than Monthly More than Daily More than monthly weekly daily

How often do you receive text messages from your doctors?

0 1 2 3 4 5

Never Less than Monthly More than Daily More than monthly weekly daily

How often do you send text messages to your doctors?

0 1 2 3 4 5

Never Less than Monthly More than Daily More than monthly weekly daily

How often do you receive phone calls?

0 1 2 3 4 5

Never Less than Monthly More than Daily More than monthly weekly daily

How often do you make phone calls?

0 1 2 3 4 5

Never Less than Monthly More than Daily More than monthly weekly daily

How often do you use your phone to take photographs?

0 1 2 3 4 5

Never Less than Monthly More than Daily More than monthly weekly daily

How often do you use your phone to browse the internet?

0 1 2 3 4 5

Never Less than Monthly More than Daily More than monthly weekly daily

How often do you download apps (applications) on a smartphone?

0 1 2 3 4 5

Never Less than Monthly More than Daily More than monthly weekly daily

Are you able to find an Australian website that provides information about depression?

О Yes О No

How often do you use the internet to look up health-related information?

0 1 2 3 4 5

Never Less than Monthly More than Daily More than monthly weekly daily

How often do you use the MS Office, e.g., MS Word, Excel or PowerPoint?

0 1 2 3 4 5

Never Less than Monthly More than Daily More than monthly weekly daily

**Section VII - Navigation**

How often do you use the internet to find out the phone numbers, addresses and opening hours of local businesses, e.g., supermarket, library, restaurant, pharmacy or GP?

0 1 2 3 4 5

Never Less than Monthly More than Daily More than monthly weekly daily

How often do you use the internet to plan your trips, including public transport options, departure times and arrival times?

0 1 2 3 4 5

Never Less than Monthly More than Daily More than monthly weekly daily

How often do you use your phone as a GPS or map?

0 1 2 3 4 5

Never Less than Monthly More than Daily More than monthly weekly daily

Section VIII – Online footprint

Do you have your own online blog?

О Yes О No

How often do you maintain your online blog?

0 1 2 3 4 5

Never Less than Monthly More than Daily More than monthly weekly daily

How often do you visit internet forums or discussion boards?

0 1 2 3 4 5

Never Less than Monthly More than Daily More than monthly weekly daily

If so, how often do you engage in conversations on internet forums or discussion boards?

0 1 2 3 4 5

Never Less than Monthly More than Daily More than monthly weekly daily

**Section IX – Vocational activities and current affairs**

How often do you engage in any self-development study activities online?

0 1 2 3 4 5

Never Less than Monthly More than Daily More than monthly weekly daily

How often do you read newspapers online?

0 1 2 3 4 5

Never Less than Monthly More than Daily More than monthly weekly daily

How often do you check weather forecasts online with your computer or smartphone?

0 1 2 3 4 5

Never Less than Monthly More than Daily More than monthly weekly daily

How often do you look and apply for jobs or studies online?

0 1 2 3 4 5

Never Less than Monthly More than Daily More than monthly weekly daily

Section X – Attitudes and technical assistance

In your belief, what are the advantages of using the internet? (Tick one or more.)

О Information on most subjects.
О Powerful search engines.
О Faster and convenient communication.
О Entertainment.
О Social networking opportunities.
О Online services and e-commerce.
Other advantages: __________________
 __________________

In your belief, what are the disadvantages of using the internet? (Tick one or more.)

О Hard to judge the information’s reliability.
О Internet-surfing can be additive and time-consuming.
О Personal and sensitive information can be stolen online.
О Pornography.
О Businesses set up traps on the internet to take advantages of people.
О Government agencies or other unknown parties may spy on your internet activities.
Other disadvantages: __________________
 __________________

How often do you look for help to use the internet?

0 1 2 3 4 5

Never Less than Monthly More than Daily More than monthly weekly daily

How often do you look for help to use a computer?
0 1 2 3 4 5

Never Less than Monthly More than Daily More than
 monthly weekly daily

How often do you look for help to use a smartphone?
0 1 2 3 4 5

Never Less than Monthly More than Daily More than
 monthly weekly daily

Who/where do you seek help to use internet/computer/smartphone?

__________________

Without internet, how often would you experience significant problems in your day-to-day life?
0 1 2 3 4 5

Never Less than Monthly More than Daily More than
 monthly weekly daily

Without computer, how often would you experience significant problems in your day-to-day life?

0 1 2 3 4 5

Never Less than Monthly More than Daily More than
 monthly weekly daily

Without smartphone, how often would you experience significant problems in your day-to-day life?

0 1 2 3 4 5

Never Less than Monthly More than Daily More than monthly weekly daily

To what extent do you agree that internet being involved in your treatment?

 0 1 2 3

Strongly Disagree Agree Strongly
 disagree disagree

To what extent do you agree that computer being involved in your treatment?

 0 1 2 3

Strongly Disagree Agree Strongly
 disagree disagree

To what extent do you agree that smartphone being involved in your treatment?

 0 1 2 3

Strongly Disagree Agree Strongly
 disagree disagree

In your opinion, do you agree that internet helps you interact/socialise more?

 0 1 2 3

Strongly Disagree Agree Strongly
 disagree disagree

In your opinion, do you agree that computer helps you interact/socialise more?

 0 1 2 3

Strongly Disagree Agree Strongly
 disagree disagree

In your opinion, do you agree that smartphone helps you interact/socialise more?

0 1 2 3

Strongly Disagree Agree Strongly
 disagree disagree

In your opinion, do you agree that internet makes your symptoms worse?

 0 1 2 3

Strongly Disagree Agree Strongly
 disagree Agree

In your opinion, do you agree that computer makes your symptoms worse?

 0 1 2 3

Strongly Disagree Agree Strongly
 disagree Agree

In your opinion, do you agree that smartphone makes your symptoms worse?

 0 1 2 3

Strongly Disagree Agree Strongly
 disagree Agree

To what extent are you interested in receiving text messages and/or emails in regards to appointment reminders, medications and symptoms?

 0 1 2 3

Not at all Somewhat Interested Very
 interested interested interested
